# Cementless Compositions for the Restoration of White Stone Based on Lime Binder and Silicon Dioxide

**DOI:** 10.3390/ma17010105

**Published:** 2023-12-25

**Authors:** Elizaveta Repina, Lyubov Zakrevskaya, Yuri Panov

**Affiliations:** 1 Department of Building Manufacture of Vladimir State University Named after Alexander and Nikolay Stoletovs (VLSU), 600000 Vladimir, Russia; lvzak@mail.ru; 2 Department of Chemical Technologies of Vladimir State University Named after Alexander and Nikolay Stoletovs (VLSU), 600000 Vladimir, Russia; tpp_vlgu@mail.ru

**Keywords:** limestone, silicon dioxide, potassium glass, ceramic microgranules, plasticizer

## Abstract

The condition of architectural monuments is directly influenced by various factors, such as anthropogenic, natural, and technological, leading to the degradation of both structures and construction materials, ultimately resulting in irreversible damage. These factors diminish the quality of construction materials, contributing to alterations and the destruction of the appearance and structure of white stone. The primary objective of this research is to develop cement-free compositions for restoring ancient masonry materials. Tests were conducted at the BM Department of VlSU using modern equipment. New material compositions for restoration have been devised, combining lime and silicon dioxide with chemical additives to enhance adhesive and strength properties. Adhesive strength increases by 1.5 times with the addition of white soot (silicon dioxide). Moreover, the incorporation of silicon dioxide positively impacts compressive strength (from 10.6 to 15.6 MPa), surpassing the strength of composites without white soot by 1.5 times. XRD confirms the developed composite’s similar phase composition to white stone, supported by optical microscopy and SEM results. Restoration composites based on these compositions exhibit homogeneous structures compatible with white stone and demonstrate high adhesion levels. These results make the proposed compositions promising for white stone restoration, ensuring structural and chemical compatibility with the restored surface.

## 1. Introduction

In the historical practice of construction, a significant number of cultural heritage sites have emerged, requiring comprehensive restoration. Therefore, the preservation of cultural heritage objects is a crucial aspect in shaping the modern appearance of historical cities. For the production of cement-free composite materials, the experience of ancient builders is used, who used materials such as lime, clay, and sand to produce strong and durable solutions.

The production of non-cementitious composite materials draws upon the ancient builders’ experience, who utilized materials such as lime, clay, and sand to create robust and durable mortars. Addressing the preservation of the historical appearance of buildings and structures, researchers pose questions. In the course of their investigations, exemplified by the analysis of four buildings, they scrutinize plastering mortars. Through optical microscopy and examination of chemical and mineralogical characteristics, they affirm that these plasters are in a deteriorated state due to the natural aging of the building and the lack of proper monitoring.

This topic remains pertinent today, as evidenced by the work of another study. Through an examination of façade plasters subjected to aggressive environments, the study demonstrates the impact of such conditions on binding agents, resulting in efflorescence, cracks, and the proliferation of fungi and mold, all of which are detrimental to architectural monuments.

The primary requirement imposed on a restoration composition is its maximum compatibility with the restored surface, as well as a shared genetic origin with the material matrix. To select a composition that is not only operationally suitable but also structurally analogous, a comprehensive analysis of the surface must be conducted. The issue of preserving the historical appearance of buildings and structures has been raised by Markssuel T. Marvila and others. In their research involving four buildings, they examine plaster solutions, affirming that through the examination of samples using optical microscopy and chemical and mineralogical analysis, it is possible to form an understanding of the structure and chemical constituents of the matrix to create an identical material.

The primary challenge in preserving cultural heritage objects is the natural aging of buildings and the lack of proper surveillance, leading to the complete deterioration of materials [1].

Various forms of influence, such as thermal and ultrasonic, disrupt the surface structure of architectural monuments, exacerbated by the age of the structures. The relevance of this topic is confirmed in the work of Afonso Rangel Garcez de Azevedo and others, where plaster solutions on building facades are analyzed under the influence of aggressive environments. The result of this impact on binding agents includes efflorescence, cracks, the proliferation of fungi, and mold, which are deleterious to architectural monuments [2].

One of the first binders used in construction, along with unburned clay, is lime. For the first time, lime was widely used in Greece. In the Mycenaean period (XVI-XI centuries BC), lime was used for plastering and facing works, as well as a grate for wall painting. Then, it began to be used in the construction of hydraulic structures [3].

For a long time, white stone was the main building material and had great historical significance, symbolizing the power and ideology in Russia during the XI-XV centuries [4].

In the pre-Mongol years, 95% of structures on the territory of the Suzdal and Vladimir lands were built exclusively of white stone [4]. A common feature of all Russian solutions of the pre-Mongolian period is their high fat content: the amount of binder is usually 50% of the solution, reaching 70% and 80%, and the lowest lime content is 30%. The composition is everywhere: calcareous or clay–lime, occasionally lime–clay [5].

After the Mongol invasion, from the end of the XI-II century to the end of the XV century, in Novgorod, limestone was still used as the main material for construction, but brick was used for more complex elements such as arches and openings [6].

The works of B. S. Shvetsov and V. S. Surovtsev clearly show the tendency to use lime in Russia during the construction of the Tithe Church in Kiev in 990. The solutions consisted mainly of crushed limestone, tsemyanka, sand, and clay. In later solutions, specially baked clay is replaced with baseboards, and powdery limestone is used. The author of [7] states that the use of crushed limestone improves the framework of the solution and creates additional crystallization centers that increase the strength. According to V. N. Jung in the XI-XV centuries. in Russia, the use of lean lime in buildings of the XV century, for example, during the construction of the Ivangorod fortress, testified to the ability to use binders obtained from limestones with an admixture of clay, i.e., the hydraulic lime.

The study of the use of natural stones in construction is also presented in the works of K. Raju and S. Ravindhar. Their work provides detailed information about the natural stones used in the construction of cultural heritage objects, about positive trends and problems, taking into account the characteristics of natural stones, such as marble, granite, limestone, etc. [8].

In the work of K. N. Nosov, construction solutions for a number of monuments of defensive architecture were studied. Masonry solutions of Russian fortresses of the XVI-XVII centuries were studied. The conclusion of the study was that all the studied solutions are calcareous–sandy. The binding mass is calcareous, rarely calcareous clay; the aggregate is quartz sand (SiO_2_), and sometimes there is clearly unburned limestone. The amount of carbonates does not exceed 10% by weight of the total aggregate [9].

Limestone gained popularity during the period of white stone architecture due to the simplicity of its production, which significantly reduced the construction time [6]. In the central part of Russia, monuments are composed mainly of hewn limestone; the composition of solutions varies due to the use of different aggregates, depending on the region and year of construction. For example, in some cases, especially in monuments of the late XII century, there is little sand in the aggregate but a large amount of clay particles. The amount of clay particles is up to 24% of the total solution. The main component of solutions at the Adimiro-Suzdal monuments is sand-up to 9.0%. Tsemyanka is represented by lean, slightly burnt clay; it is very likely that this impurity got into the solution together with lime. The solutions of the Monomakhov Cathedral in Suzdal have a different character: the binder here is 55%, the main component in the aggregate is tsemyanka (64% of the aggregate), the alder amount of clay particles is almost 33%, and a small amount of sand [10].

To obtain a long-lasting and high-quality restoration composition, it is necessary to study the properties of authentic materials of architectural monuments. Therefore, the restoration process involves many interrelated stages, the first of which is analysis [11]. As a result of which, a decision is made to strengthen or completely replace masonry elements. Not only are the strength characteristics studied, but special attention is also paid to the study of the microstructure and chemical composition since, on the basis of the obtained data, a selection of components of the restoration composite is created [12].

The main distinguishing feature of limestone from cement stone is the advantage of reducing greenhouse gas emissions as a result of absorbing carbon dioxide over time. In the future, there will be a process of carbonation and strength gain due to the absorption of atmospheric carbon dioxide, so limestone and lime mortars have been used since ancient times [13].

On the basis of all the above, it is necessary to solve the problem of forming an authentic structure with white stone, that is, with the ratio of crystalline and amorphous phases identical to white stone. The principle of affinity of structures is to minimize the physical, mechanical, and structural differences between the created pre-composite composition and the restored surface [14]. This is achieved by establishing chemical bonds between various raw materials forming the composite. When choosing the main components for creating a restoration material, special attention is paid to the possibility of its collaboration with the main materials of the structure [15].

To create a long-lasting contact, it is necessary to create a stable internal bond [16]. Therefore, the principle of affinity of structures is to minimize the physical, mechanical, and structural differences between the created precomponent composition and the restored surface. This is achieved due to the ratio of the components that form the corresponding crystal lattices. This contact zone should have the same basic properties as the matrix of the material [17]. The structure of the main and developed composite should be a single monolithic layer with the same physical and technical characteristics, including the coefficient of thermal expansion. Coincide in macro- and microstructure, bio-, and weather resistance [18,19].

To accurately select an authentic composition, it is necessary to fully examine the characteristics of the existing masonry, the degree of damage, as well as the condition of the structure as a whole. As a result of the restoration process, there should be no interference with the existing aesthetic value of the masonry, and its structural integrity should be preserved [16].

Now, the problem of preserving historical heritage is one of the leading places in modern construction. In an aggressive urban environment, weathering and destruction of natural stone occurs. Over time, a layer of dirt, dust, efflorescence forms on the stone, and fungal mycelium forms [20].

Emissions of SO_2_ into the atmosphere and sulfates from natural sources are an integral part of the life process, which interact with lime masonry, causing significant damage to the white stone. This is called cysuulfate-induced decomposition, which occurs by the formation of insoluble Ca^2+^ salts, and as a result of the reaction of CaCO_3_ with SO_2_ or acid rain, a “gypsum crust” is formed. «formed when sulfuric acid (H_2_SO_4_) is exposed from the atmosphere, the products of this reaction are most often formed by semi hydrate (CaSO_4_·0.5H_2_O) and gypsum (CaSO_4_·2H_2_O) depending on relative humidity [21]».

From a scientific and historical point of view, to prolong the life of cultural heritage sites, it is advisable to use hydrated lime as the main component since the main task of restoration is to recreate identical compositions for white stone [22]. Additional components are selected depending on their chemical nature and physical and mechanical properties, which will create a high-quality composition for the restoration of white stone masonry. Therefore, it is necessary to determine the porosity and pore distribution, water absorption, and thermal expansion, as well as the mechanical properties of the rock. The material must also have similar optical properties [23].

Summarizing the above, we can say that the most important thing for restoration is that the old and new materials are compatible [24]. Even at their border, moisture and mechanical stresses will accumulate, which will eventually lead to a weakening of strength and the release of new material. Such processes, as a rule, lead to the destruction of the original, which is very dangerous during restoration work [25].

## 2. Materials and Methods

The tests were carried out on the basis of the Department of the Building Manufacture (BM) of the Alexander Grigoryevich and Nikolai Grigoryevich Stoletov (VlSU), and standard methods corresponding to GOST R 57921-2017 “Polymer composites. Test methods. General requirements” [26] were used to determine the physical and chemical characteristics. To determine the strength indicators and mechanical characteristics of the compositions, the methods specified in GOST R 58527-2019 “Wall materials. Methods for determination of ultimate compressive and bending strength” [27].

The main part of the tests was carried out on control samples of dimensions 60 × 80 × 20. To test the compressive strength, cubic samples with dimensions of 20 × 20 × 20 mm were used. In the conducted experiments, modern equipment and devices were used, such as the P-50 press (OOO RSCIM, Neftekamsk, Russia), the heat and cold testing chamber, SM-60/150-80-TX (vol: 80 L, temp: from −60 °C) (SM Climat, St. Petersburg, Russia), material moisture meter VIMS-2.12 (Interpribor, Chelyabinsk, Russia), and the strength meter of building materials IPS-MG4.03 (OOO SKB Stroypribor, Chelyabinsk, Russia), digital microscope Levenhuk D320L (OAO Levenhuk, St. Petersburg, Russia).

For the investigation of the microstructure of the white stone and the developed non-cementitious composition, a FEI QUANTA 3D 200I scanning electron microscope (manufacturer: FEI Company, Hillsboro, OR, USA) equipped with an Everhart–Thornley secondary electron detector was employed. The accelerating voltage ranged from 5 to 10 kV, with a beam current ranging from 46 pA to 0.12 nA.

The mineralogical composition was studied by X-ray phase analysis using a powder diffractometer Bruker D8 ADVANCE (manufacturer: Bruker Corporation, Billerica, MA, USA). The Bragg–Brentano geometry with CuK radiation was used to survey the samples, and X-rays were recorded using a position-sensitive Lynx-eye detector (Bruker Corporation, Billerica, MA, USA)). DIFFRAC. SUITE V.2.0 software was used for data analysis, and Powder Diffraction File PDF 2 was used as a database for mineral identification Diffraction File PDF2. The survey was carried out in an X-ray tube with a power of 1.6 kW, a voltage of 40 kV, and a current of 40 mA. The properties of the identified phases in the investigated materials were obtained through phase analysis in the DIFFRAC.EVA V.2.0 software. The visualization of the crystal structures of the identified phases was achieved using the VESTA Ver. 3.5.8 software.

The synthesized formulations were tested for frost resistance by the accelerated method, using a 5% solution of sodium chloride (sodium chloride). Initially, it was necessary to saturate the samples with a solution, then they were subjected to freezing for 2.5 h at a temperature of −20 °C, and then they were defrosted for 3.5 h at a temperature of +20 °C. After completing the required number of freezing and thawing cycles, the samples were subjected to compression tests. The remaining samples were tested for water resistance: they were saturated with water, after which they were subjected to a compression test. Using the softening coefficient, the water resistance of the obtained material was estimated according to GOST 9128-2013 “Asphaltic concrete and polimer asphaltic concrete mixtures, asphaltic concrete and polimer asphaltic concrete for roads and aerodromes. Specifications” [28], and GOST 13905-2005, “Glass containers. Method of testing the water resistance of inner surface” [29].

For result reproducibility, each composition was synthesized a minimum of three times with a component increment of 0.5%.

The density and water absorption by mass were calculated for the samples; for this purpose, the volume of samples was initially determined using measuring instruments, and their mass was determined using laboratory scales.

## 3. Results

The selection of materials for restoration is a paramount consideration, as the use of materials divergent from those originally employed in construction can lead to compatibility issues between the restored surface and the developed composite. Parameters such as deformability, strength, thermal expansion, and chemical compatibility must be carefully considered. The primary drawback of cementitious mortar lies in its excessive porosity and incompatibility with white stone due to differing coefficients of thermal conductivity, resulting in the deterioration and cracking of masonry. Additionally, the production of cement releases a significant amount of carbon dioxide, rendering this material environmentally unsuitable. Therefore, in restoration efforts aimed at emulating white stone, it is advisable to utilize lime binding, marble chips, and microdolomite, along with various chemical additives. These additives include liquid potassium silicate, silicon dioxide, the plasticizer Mefluxs, and foam glass ceramic microgranules. The chemical reactions involving lime and raw materials lead to the formation of calcium and magnesium carbonates and hydrosilicates, as evidenced by XRD. These components are environmentally justified, as their production does not release harmful compounds or other environmentally unsafe elements into the atmosphere.

All the studied components and their properties considered in this study are included in Table 1.

**Figure 1:**
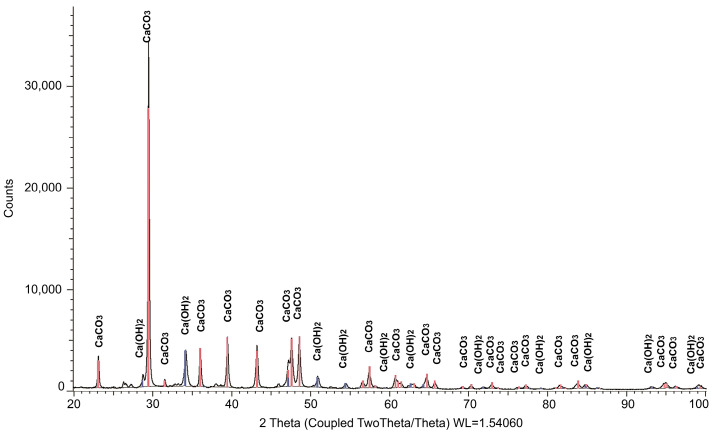
Results of the study of the mineralogical composition of hydrated lime (illustration by the authors).

**Figure 2:**
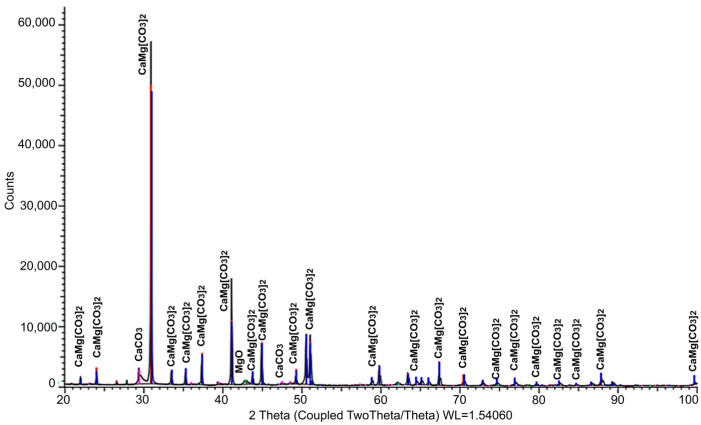
Results of the study of the mineralogical composition of microdolomite (illustration by the authors).

**Figure 3:**
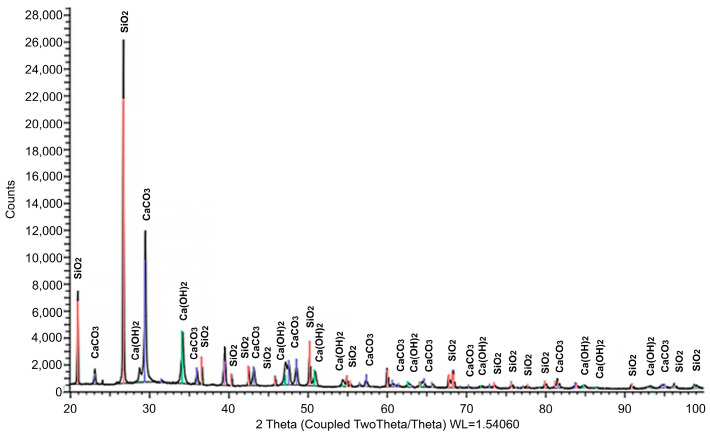
Results of the study of the mineralogical composition of white soot (silicon dioxide) (illustration by the authors).

The mineralogical composition was studied by X-ray phase analysis using a powder diffractometer Bruker D8 ADVANCE (Bruker Corporation, Billerica, MA, USA). The results obtained indicate the presence of calcite and portlandite crystals in the initial materials. The results of XRD analysis of the mineral composition of the main raw material components are presented in Figure 1, Figure 2 and Figure 3. The identified phases corresponding to the peaks are marked on the diffractograms. In Figure 1, the presence of two phases is observed: CaCO_3_ and Ca(OH)_2_, with the predominance of the former. Figure 2 demonstrates the identification of three phases: the predominant CaMg[CO_3_]_2_, along with small amounts of MgO and SiO_2_. The analysis results in Figure 3 revealed four phases: SiO_2_, CaCO_3_, CaMg[CO_3_]_2_, and Ca(OH)_2_, with a relatively low quantity of the latter. Table 2 summarizes the results of the quantitative and qualitative composition of raw materials components.

**Figure 4:**
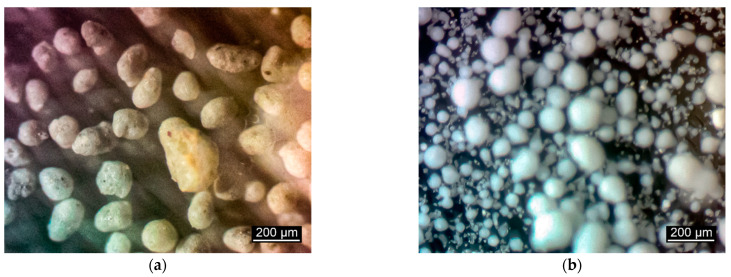
(**a**) Results of studying the microstructure of ceramic foam granules of “KERWOOD”, (**b**) Results of studying the microstructure of silicon dioxide.

Table 3 presents the experimental formulations for the restoration of the white stone masonry. The results of studying the microstructure of raw components are shown in Figure 4 (magnification ×100).

Table 3 shows experimental compositions for the restoration of white stone masonry.

The main component of the developed formulations is hydrated lime with corrective strengthening additives. Microcrystalline granules, due to their physical and technical properties, increase the fire resistance of materials and reduce their specific weight, easing the load on structures. In particular, silicon dioxide for creating a strong crystal lattice (white carbon black BS120) allows for the improvement of the mechanical characteristics of the composition due to the formation of calcium and magnesium hydrosilicates as a result of the development of the Pozzolan [30]. The ultrafine pozzolanic additive in the form of amorphous silica possesses a developed surface and, simultaneously, high surface energy, accompanied by significant pozzolanic activity, thereby leading to enhanced performance properties when applied reaction [31].

Table 4 presents the averaged results from three measurements of the study on the physico-mechanical properties of the proposed compositions and natural stone.

From the table, it is evident that the presence of silicon dioxide in the composition enhances its strength characteristics, obviously due to a more complete development of the Pozzolan reaction between the components, with the formation of a strong crystal structure Equations (1) and (2).

SiO_2_·nH_2_O + Ca(OH)_2_ → nCaH_2_SiO_4_·H_2_O, (1)

Ka_2_O·2SiO_2_ + CaO → Ka_2_O·SiO_2_ + CaSiO_3_ (2)

As a result of the reaction occurring between calcium hydroxide (Ca(OH)_2_) and amorphous silica dioxide (SiO_2_·nH_2_O), a gel of calcium silicate hydrate (C-S-H) is formed. This gel fills the pores of the stone, positively influencing its strength characteristics. Compositions utilizing amorphous silica exhibit strength values 1.5 times higher than those of composites without the additive. Frost resistance studies, as presented in the table, indicate that the developed composite, like the white stone, withstands an equal number of alternate freeze–thaw cycles, indicating comparable durability.

**Figure 5:**
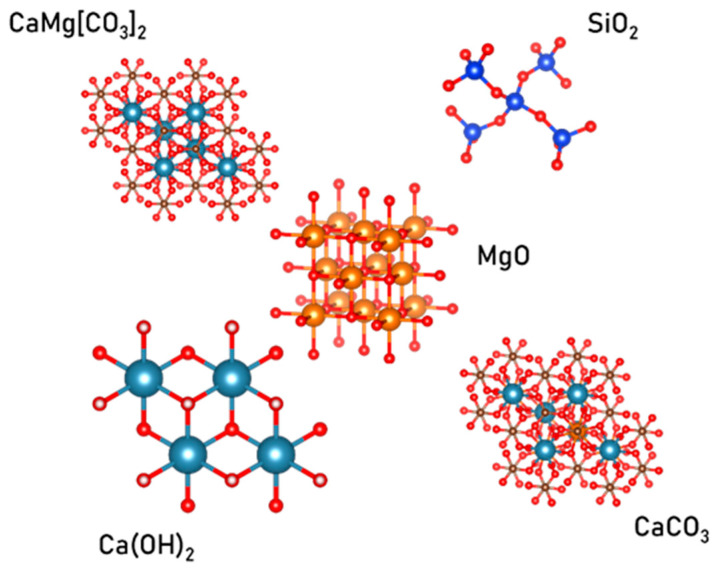
Crystal structures of the identified phases.

As a result of comparing the samples based on their physico-mechanical properties, positive outcomes of adding silicon dioxide can be inferred. Consequently, the crystal structures of the identified phases are illustrated in Figure 5.

**Figure 6:**
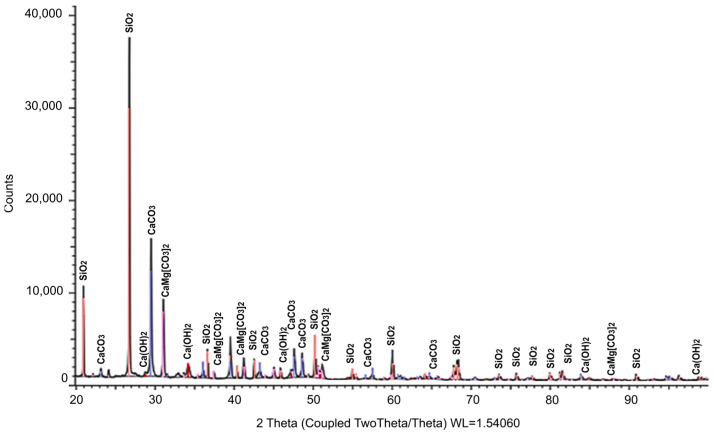
Results of the study of the mineralogical composition of white stone (illustration by the authors).

**Figure 7:**
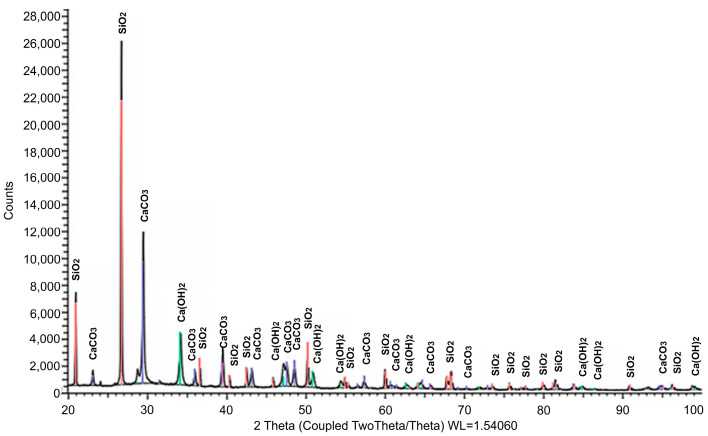
Results of the study of the mineralogical composition of the developed composite.

Figure 6 and Figure 7 show the results of the study of the mineralogical composition of white stone and the developed composite. During XRD analysis of the compositions, phases of SiO_2_, CaCO_3_, and Ca(OH)_2_ were identified. Table 5 summarizes the results of quantitative and qualitative composition, respectively.

A comparison of the results of chemical analysis and mineralogical compositions of composites based on white soot (silicon dioxide) and lime binder with ancient limestone showed the identity of the synthesized and restored material (Table 5).

**Figure 8:**
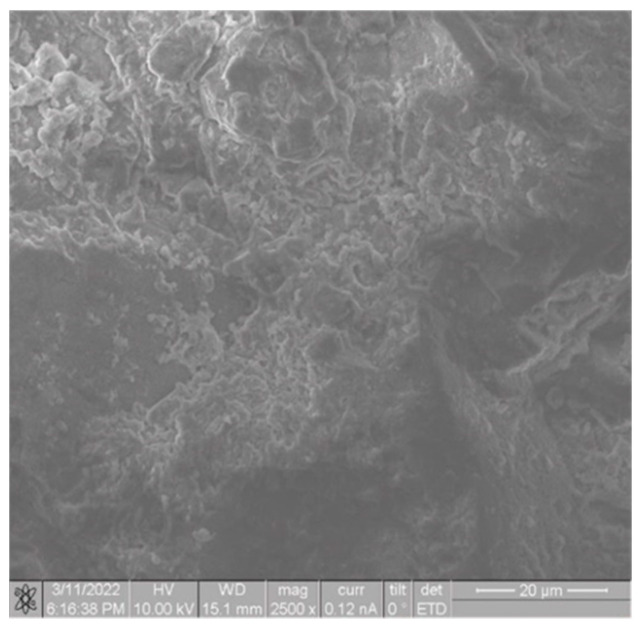
SEM of the source material (authors’ illustration) magnification ×2500.

**Figure 9:**
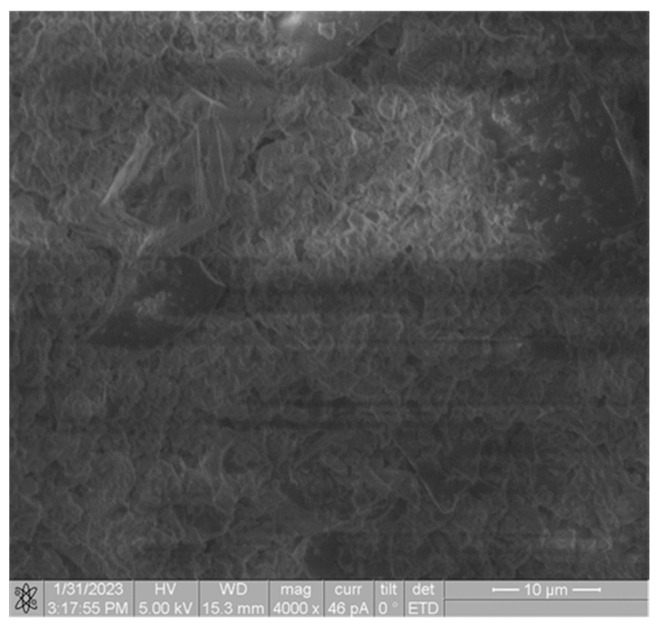
SEM of the developed cement-free restoration composition (authors’ illustration) magnification ×4000.

The microstructure indicates the presence of a well-developed spatial crystalline structure, consisting of needle-like formations forming an elaborate three-dimensional network (Figure 8 and Figure 9), as evidenced by the results of mechanical property studies: for white stone, ranging from 6 to 36 MPa, and for the proposed composite, from 10.6 to 15.6 MPa.

**Figure 10:**
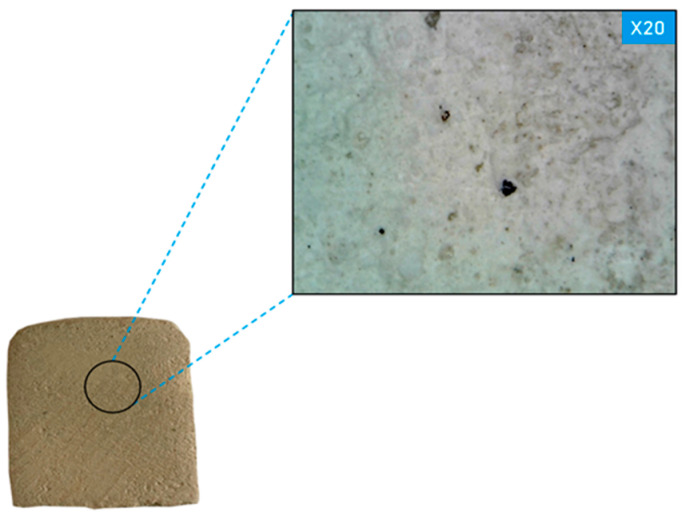
Contact area of the source material and the developed cement-free restoration composition (illustration by the authors).

Figure 10 shows the contact zone of the white stone precomponent composition according to the results of optical microscopy, which shows that the precomponent composition is a related structure to the white stone, only with fewer pores and voids.

Analysis of the results of studying the physical and mechanical properties allows us to conclude that the composition corresponds to the strength characteristics of limestone and has a lower density than that of white stone, which allows it to be used as a pre-composite composition, taking into account the ease of construction.

## 4. Discussion

The presented study explores critical considerations associated with the selection of materials for restoration, underscoring the pivotal role of compatibility between original construction materials and the developed composite. The examination encompasses potential challenges linked to the utilization of heterogeneous materials, notably cementitious mortar, addressing issues such as excessive porosity and incompatibility with white stone, leading to the deterioration of masonry and the development of cracks. Environmental concerns associated with cement production underscore the imperative for alternative restoration materials.

In response to these challenges, the research advocates for a restoration approach utilizing lime binding. The utilization of lime as the primary component enables maximal adhesion to the restored surface. The introduction of amorphous silicon dioxide as an ultrafine pozzolanic additive emerges as a crucial element, enhancing the operational characteristics of the composite by establishing a robust crystalline structure. Chemical bonds are fortified through the inclusion of silicon dioxide, resulting in a densely uniform structure and heightened fire resistance and heat resistance.

The incorporation of chemical additives such as potassium glass, marble chips, and plasticizers enhances component adhesion and material mechanical strength. Comprehensive mineralogical analyses, including XRD analysis and microstructural investigations, furnish valuable insights into the synthesized materials and their compatibility with ancient limestone.

Survey results indicate that, for the restoration of architectural monuments, it is preferable to employ building materials similar to those originally used in the construction [32]. Restoration compositions should not surpass the main material in strength but should correspond to it in structure and appearance. A notable aspect of the study involves a meticulous comparison between the developed composite and ancient limestone, emphasizing the identity of the synthesized and restored material.

Upon studying limestone, structural weakening in the surface zone of historical material due to external loads and atmospheric influences was identified. Restoration necessitates measures to strengthen the structure by applying hydrophobic agents that impede moisture penetration into the stone [33].

To address the aforementioned issue, rock type, water absorption, and strength were studied and compared with the developed composite material. As a result of this work, cementless pre-compounding compounds were formulated for the restoration of white stone, exhibiting strength characteristics comparable to the ancient material. They possess similar structures, wherein the ratio of amorphous and crystalline phases mirrors that of white stone, facilitating a high level of adhesion and the potential for complete replacement of missing masonry fragments.

The proposed composition, characterized by high thixotropy and plasticity, enables surface shaping with deviations from the system plane. Developed composite compositions for the restoration and finishing of buildings exhibit adhesive strength (R_adg_) of 0.65 MPa, compressive strength (R_s_) ranging from 10.6 to 15.6 MPa, water absorption by weight at 11–12 wt.%, and porosity of 10–12%.

The potential for enhancing the durability of lime-based coatings through the introduction of an additive containing amorphous silicon dioxide is substantiated. Patterns of structure formation in a lime composite in the presence of an additive are disclosed, involving the formation of calcium hydrosilicates and an increase in the amount of chemically bound lime.

It was observed that the introduction of an additive based on amorphous silicon dioxide into the lime mixture formulation accelerates coating curing. Additionally, the introduction of an additive based on silicon dioxide into the lime–sand composition contributes to an increase in compressive strength after 28 days of air-dry hardening.

A primer composition for preliminary strengthening has been developed, including potassium glass, which induces a calcium-induced effect through the formation of calcium silicate. From an aesthetic perspective, the composition maintains the appearance and preserves the individuality of architectural monuments.

In conclusion, this study provides a valuable contribution to the development of advanced restoration materials, demonstrating the efficacy of lime-based compositions with additions of amorphous silicon dioxide. A comprehensive analysis and a systematic approach lay the foundation for further advancements in the fields of cultural heritage preservation and construction materials science.

## Figures and Tables

**Table 1:** Basic properties of raw materials.

Component	Properties			
	Bulk Density, kg/m^3^	Humidity, %, Max	pH Value	Mass Fraction of Oxides, %
Microdolomite	1200	0.3	8–9	-
Marble chips MK500	2710	0.2	8–10	55.2 (CaO)
Liquid potassium glass HISOL	1050	-	2–3.5	-
Foam ceramic microgranules «KERWOOD»	480	0.5	-	-
Hydrated lime	1082	5	12–12.5	>67 (CaO + MgO)
Silicon dioxide (White Carbon Black BS120)	180	6.5	-	87 (SiO_2_)

**Table 2:** Results of quantitative and qualitative composition of raw materials components.

Sample	Phase	Quantity, %	Space Group	Lattice Parameters		
				Type	*a*, nm	*c*, nm
Hydrated lime	CaCO_3_	90.60	$R\bar{3}c$ (167)	hex.	4.9844	17.0376
	Ca(OH)_2_	9.40	$R\bar{3}m1$ (164)	hex.	3.5890	4.9110
Microdolomite	CaMg[CO_3_]_2_	49.22	$R\bar{3}$ (148)	hex.	4.8120	16.0200
	CaMg[CO_3_]_2_	48.09	$R\bar{3}$ (148)	hex.	4.8079	16.0100
	MgO	0.53	$Fm\bar{3}m$ (225)	cub.	4.2170	4.2170
	SiO_2_	0.37	P3_2_21 (154)	hex.	4.9115	5.4038
White soot (silicon dioxide)	SiO_2_	44.12	P3_2_21 (154)	hex.	4.9115	5.4038
	CaCO_3_	29.41	$R\bar{3}c$ (167)	hex.	4.9844	17.0376
	Ca(OH)_2_	26.47	$R\bar{3}m1$ (164)	hex.	3.5890	4.9110

**Table 3:** Cement-free sections for restoration of white stone masonry.

Brand of Composition	Components, Mass %							
	Hydrated Lime	Marble Chips MK500	Liquid Potassium Glass HISOL	Microdolomite	Plasticizer Mefluks Plasticizer	Mefluks Silicon Dioxide (White Carbon Black BS120)	Microscopic Microgranules 0.1–0.3 mm	H_2_O
IHD-1	32.6	14.5	2	8.9	0.2	3.8	6.9	31.1
IHD-2	32.6	12.7	2	7.5	0.2	6.8	5.6	32.6
IHD-3	32.6	10.5	2	6.3	0.2	9.8	4.3	34.3
I-1	35.4	15.8	2	11.8	0.34	0	8.7	25.9
I-2	32.5	16.2	2	12.4	0.34	0	9.3	27.3
I-3	29.3	17.4	2	14.3	0.3434	0	10.2	26.5

**Table 4:** Results of the study of physical and mechanical properties, proposed compositions, and natural stone.

Brand of Composition	Name of Properties					
	Frost Resistance, Cycles	Compressive Strength, R_sj_, MPa	Porosity, %	Water Absorption, %	Density, kg/m^3^	Adhesive Strength R_adg_, MPa
Natural stone	75	6–36	4–26	5–15	1850–2200	-
IHD-1	75	10.6	10	11	1190	0.65
IHD-2	75	12.7	10	12	1250	0.64
IHD-3	75	15.6	12	11	1280	0.65
I-1	75	9.8	9.8	6.5	1350	0.36
I-2	75	7.5	10	6	1380	0.37
I-3	75	7.8	11	6.5	1290	0.4

**Table 5:** Results of quantitative and qualitative composition of white stone and developed composite.

Sample	Phase	Quantity, %	Space Group	Lattice Parameters		
				Type	*a*, nm	*c*, nm
White stone	SiO_2_	52.50	P3_2_21 (154)	hex.	4.9115	5.4038
	CaCO_3_	24.62	$\bar{R3}c$ (167)	hex.	4.9844	17.0376
	CaMg[CO_3_]_2_	19.67	$\bar{R3}$ (148)	hex.	4.8079	16.0100
	Ca(OH)_2_	3.21	$R\bar{3}m1$ (164)	hex.	3.5890	4.9110
Developed composite	SiO_2_	56.78	P3_2_21 (154)	hex.	4.9115	5.4038
	CaCO_3_	31.08	$R\bar{3}c$ (167)	hex.	4.9844	17.0376
	Ca(OH)_2_	12.14	$R\bar{3}m1$ (164)	hex.	3.5890	4.9110

## Data Availability

Data are contained within the article.
